# Methodological and Procedural Considerations for Developing Decision Analytic Models to Assess the Health Economic Impacts of Newborn Bloodspot Screening: A Systematic Methodological Review

**DOI:** 10.3390/ijns11040096

**Published:** 2025-10-17

**Authors:** Jim Chilcott, Alice Bessey, James R. Bonham, Iván Castilla-Rodríguez, Sarah Davis, David Elliman, Sara Hunt, Chris Hyde, Silvia Lombardo, Jason Madan, John Marshall, Joan Morris, Katherine Payne, Oliver Rivero-Arias, Bethany Shinkins, Graham Shortland, Susan Spillane, Anthea Sutton, Sian Taylor-Phillips, Cristina Visintin

**Affiliations:** 1 Sheffield Centre for Health and Related Research (SCHARR), The University of Sheffield, Sheffield S1 4DA, UK; j.b.chilcott@sheffield.ac.uk (J.C.); a.r.bessey@sheffield.ac.uk (A.B.); j.bonham@nhs.net (J.R.B.); a.sutton@sheffield.ac.uk (A.S.); 2 Departamento de Ingenieria Informática y de Sistemas, Universidad de La Laguna, 38200 San Cristóbal de La Laguna, Spain; icasrod@ull.es; 3 Great Ormand Street Hospital, London WC1N 3JH, UK; david.elliman@nhs.net; 4 Alex-The Leukodystrophy Charity London, London SE15 5EB, UK; sara@alextlc.org; 5 Health and Community Sciences, The University of Exeter, Exeter EX1 2LU, UK; c.j.hyde@exeter.ac.uk; 6 UKNSC Secretariat, London SW1H 0EU, UK; silvia.lombardo@dhsc.gov.uk (S.L.); john.marshall@dhsc.gov.uk (J.M.); cristina.visintin@dhsc.gov.uk (C.V.); 7 Warwick Medical School, The University of Warwick, Coventry CV4 7AL, UK; j.j.madan@warwick.ac.uk (J.M.); bethany.shinkins@warwick.ac.uk (B.S.); s.taylor-phillips@warwick.ac.uk (S.T.-P.); 8 Population Health Research Institute, St George’s, University of London, London SW17 0RE, UK; jmorris@sgul.ac.uk; 9 Division of Population Health, Health Services Research and Primary Care, The University of Manchester, Manchester M13 9PL, UK; katherine.payne@manchester.ac.uk; 10 National Perinatal Epidemiology Unit, Nuffield Department of Population Health, University of Oxford, Oxford OX3 7LF, UK; oliver.rivero@npeu.ox.ac.uk; 11 Cardiff and Vale University Health Board, Cardiff CF14 4XW, UK; graham.shortland@wales.nhs.uk; 12 Health Information and Quality Authority (HIQA), T12 Y2XT Cork, Ireland; sspillane@hiqa.ie

**Keywords:** newborn bloodspot screening, costs and cost analysis, economic, neonatal screening, newborn screening

## Abstract

This methodological review identifies challenges in the development of health economic evaluations of newborn bloodspot screening (NBS) interventions and their consideration in NBS policy making. A systematic review of health economics methodological studies in NBS and stakeholder consultation was undertaken. The intervention under examination was defined as health economic decision analytic modelling used as decision support to NBS policy makers. An iterative search strategy was used to identify studies, and a data extraction framework was based upon a simple decision analytic model structure for the NBS decision problem. Synthesis was facilitated by two stakeholder workshops, which focused on ensuring the complete identification of challenges and developing recommendations. Sixteen methodological studies were identified. Data were extracted on challenges in decision criteria, decision variables, decision problem scope, defining model structure, selecting modelling method, the target condition, the screening test/protocol, outcome nodes, and other categories. Recommendations are made concerning supporting NBS decision making, NBS economic model structure and methods, data and estimation of model parameters, and overarching considerations. Recommendations for decision processes and methods research are put forward for the consideration of NBS policy makers and commissioners of research.

## 1. Introduction

Newborn bloodspot screening (NBS) is a complex public health programme that has the potential to provide early detection of many health conditions, enabling timely management and treatment. The variation in conditions included within NBS programmes internationally [1] reflects the challenging nature of policy making in this context. Many of the conditions for which NBS may be appropriate are rare or ultra rare, affecting less than 1 in 2000 or 50,000 people, respectively, with all that entails for knowledge, evidence, and practice development. Furthermore, the complexity of screening, the interacting components within each intervention, the interaction between intervention and setting, and the complex nature of many NBS policy questions further challenge evidence generation and policy making [2].

In the four UK countries, the National Screening Committee (UKNSC) advises ministers and the National Health Service (NHS) about all aspects of screening, including the case for introducing new conditions to the NHS Newborn Blood Spot Screening Programme, with additional committees in Wales and Scotland overseeing implementation in those countries. The UKNSC’s approach to policy development and screening practice in the bloodspot setting is described in Lombardo et al. [3] and includes evidence review, modelling, and empirical evaluation. In 2022, the UKNSC established the Blood Spot Task Group (BSTG) to identify practical and innovative approaches to help researchers and others develop evidence that could help the UKNSC make robust recommendations [4]. This methodological review was commissioned by the BSTG to examine the technical and procedural considerations for decision-analytic models of NBS interventions and to complement existing methods guidelines [5,6]. The aims of this project were to answer the following research questions: what are the challenges experienced in the development and consideration of economic evaluations of NBS interventions of the type submitted to the UKNSC? What recommendations regarding processes and methods can be made to address these challenges? And what are the research implications of these challenges?

## 2. Materials and Methods

The project comprised a systematic review of the methodological literature of health economic assessments evaluating NBS programmes and workshops with NBS stakeholders from the UK and internationally, including Spain, Ireland, and the US. Stakeholders included UKNSC members to ensure a complete identification of challenges experienced in UK policy making, whilst health professional and patient voice members ensured all stakeholder perspectives were accessed. The intervention under examination was defined as health economic decision analytic modelling used as decision support to NBS policy makers. The protocol for the study is available on PROSPERO [7].

An iterative ‘pearl growing’ search strategy was applied, using five initial studies [5,6,8,9,10] to develop focused search strategies (see Supplementary Material for further details). The searches were conducted during July–August 2023, updated April 2024, and concluded on saturation. Sources searched included MEDLINE and Embase via Ovid, EconLit, Tufts CEA Registry, and MATHSCINET. Supplementary searches, including reference list checking and consultation with experts, provided additional material. Inclusion criteria were methodological papers dealing with processes and methods for undertaking economic decision models of newborn bloodspot screening interventions. Exclusion criteria included single-condition case studies and reviews, and methods papers not concerning health economics or NBS. Further details are given in Supplementary Materials.

**Figure 1:**
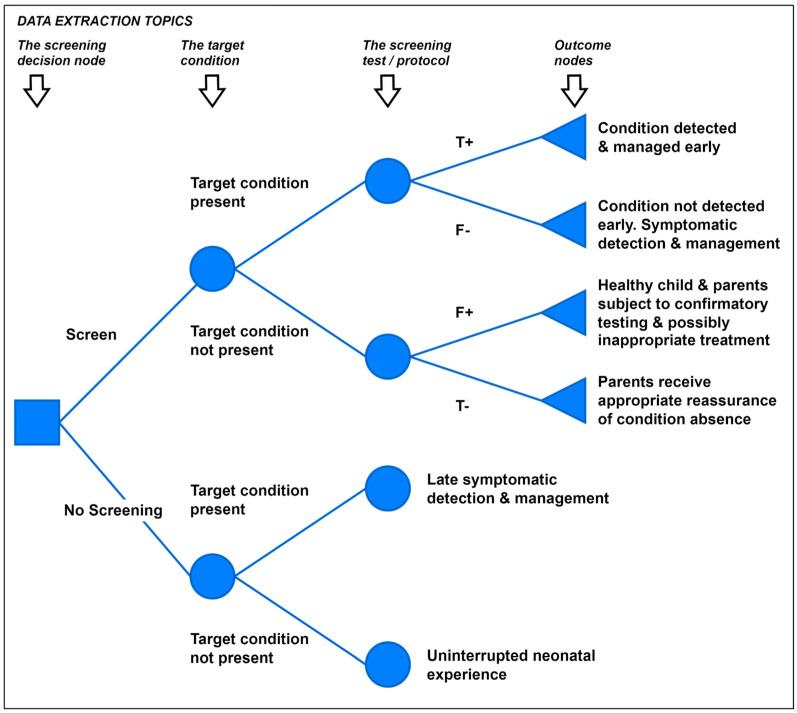
Generic simple decision-tree model for the NBS decision problem.

Data extraction focuses on the identification of challenges in NBS health economics modelling. A simple decision-analytic model structure for the NBS decision problem, Figure 1, was used to provide a framework for data extraction. An additional category to gather data on the problem scoping and structuring process was also defined, together with an ‘Other’ category to capture issues outside this a priori framework. The data extraction topics include the screening decision node, including decision criteria and decision variables; defining the decision problem scope and model structure, including selecting the modelling method; the target condition; the screening test/protocol; the outcome nodes; and other considerations. The extraction template was piloted on two of the methodology pearls [9,11] and amendments agreed with the UKNSC project team. Data extraction was undertaken by two reviewers (JC, AB); double extraction was undertaken for 30% of selected studies, with disagreements resolved by discussion.

Critical appraisal of methodological studies is challenging since there is currently no tool that covers the broad range of potentially included studies [12]. Quality was, therefore, assessed with the Scale for the Assessment of Narrative Review Articles (SANRA) instrument [13]. This instrument was developed to aid the editorial assessment of non-systematic reviews and includes six items, with a maximum score of 12 and an average score of 6.6 in a sample of accepted journal manuscripts. The data extraction domains provide an a priori framework for a “best-fit” framework synthesis. Workshops considered both the adequacy of the a priori framework and the issues identified within the framework components. Synthesis was facilitated by the two stakeholder workshops. The first workshop discussed the systematic review data extraction and provided a synthesis of the issues raised and focused on obtaining a complete identification of challenges. The second workshop completed the synthesis and focused on the development of the recommendations outlined in this paper.

## 3. Results

**Figure 2:**
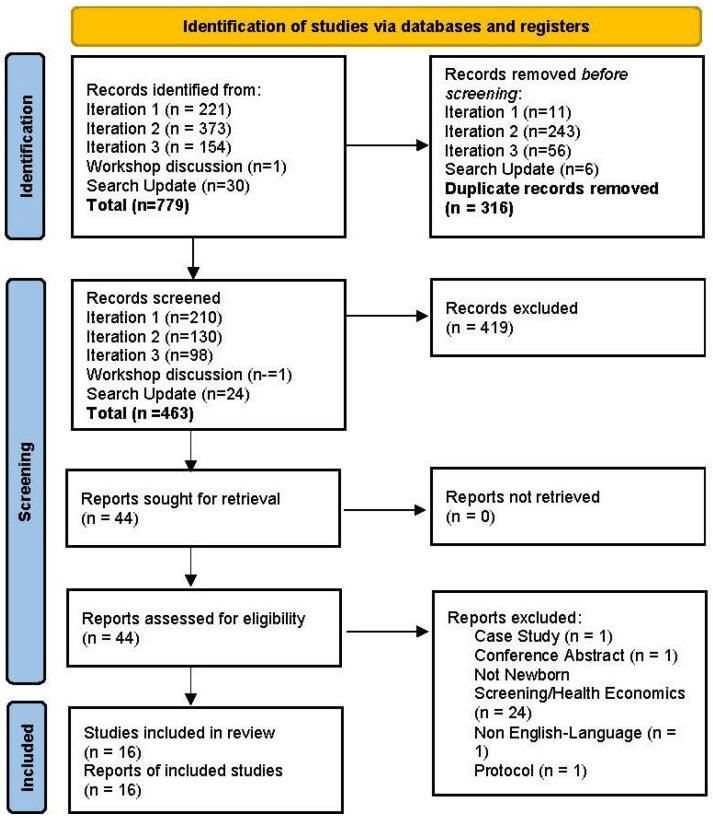
PRISMA 2020 flow diagram for new systematic reviews which include searches of databases and registers.

The searches retrieved a total of 779 references, with 16 studies included in the review following sifting. The PRISMA diagram is presented in Figure 2. The included studies comprised seven critiques based on reviews of models (SANRA average score 8) [5,11,14,15,16,17,18]; three systematic reviews (SANRA average score 11) [8,9,19]; three education and debate studies, including one book chapter (SANRA average 9) [20,21,22]; one consensus statement (SANRA 12) [6]; and two studies that described new methods developments (SANRA 12) [23,24]. A topic summary of the data extractions is presented in Table 1, together with the SANRA scores. The key findings from the review and workshops discussions are presented below, categorised on the basis of the data extraction framework previously described.

### 3.1. The Screening Decision Node

#### 3.1.1. Decision Criteria

Transparent and standardised criteria are the starting point for a rational offer of healthcare services [11]. Whilst most NBS policy making internationally shares a common ancestry in Wilson & Jungner criteria [25], there is variation in policy making practice and a growing call to reconsider their appropriateness for NBS decisions making [3,26,27]. A particular area of variation is in the consideration of economic criteria [28]. Against this background, systematic reviews of health economic analyses of NBS interventions similarly find a high degree of variation in the outcomes being reported, with less than half of studies reporting health outcomes measured in QALYs or DALYs [8,9]. This variation occurred despite recommendations by Langer et al. [6] in 2012 that economic analyses should present incremental cost-effectiveness as a primary economic measure, supported by disaggregated and aggregated costs and health outcomes to support cost-consequence analysis.

Patient voices in workshop discussion felt strongly that outcomes should adequately capture parental and family impacts, such as caring and reproductive choice, “because the parents bear all of the burden… they give permission for the test, they have to take the results… and it’s with them through their life… they’re committed”. There is, however, some international variation in the priority given to parental considerations in decision making [3]. In the UK, whilst such considerations can strengthen or weaken the economic case for screening, screening needs to demonstrate expected health benefits to the newborn who would be screened [27].

This variation in practice suggests that there is a central challenge for policy makers and researchers to develop explicit guidance on the range of health economic, health, resource, and cost outcomes that would support NBS decision making and are potentially feasible.

#### 3.1.2. Decision Variables

Decision variables relate to the context of the decision problem and include the time horizon, perspective, discount rates, and cost-effectiveness threshold.

The time horizon of an economic evaluation should capture all effects of an intervention. In NBS, this commonly implies a lifetime horizon; however, Png, in reviewing antenatal and newborn screening case studies, and Cacciatore, in reviewing newborn screening studies, identify that this was adopted in less than half of the studies and was often justified by a lack of long-term evidence [8,9]. It has been suggested that presenting outcomes for different time horizons may help decision makers understand uncertainty [29].

The perspective, important in defining outcomes within an analysis, the discount rates, and cost-effectiveness thresholds are all specific to the economic context of a decision maker’s jurisdiction. Variation between settings is therefore appropriate and to be expected. The methods literature identified that there was commonly a lack of justification given for the choice of these variables and that, since they could be significant drivers of results, generalisation between studies was often difficult [8,9,18,22]. The challenge for analysts is therefore to achieve transparency and enable comparability of economic analyses whilst satisfying the needs of decision makers. Potential solutions discussed in the workshops were the publication of extensive sensitivity analyses and consideration of developing open models.

### 3.2. Defining the Decision Problem Scope and Model Structure

#### 3.2.1. Decision Problem Scope

The scope of an economic evaluation defines the population, intervention, comparator, and outcomes. Workshop discussions identified important interactions between these concepts when considering NBS. For instance, whilst the definition of the intervention involves specification of a target condition(s), in practice the affected population is determined by the performance of the diagnostic protocol, with the conditions identified often differing from the defined target condition(s) [20]. The literature [22] and discussion both highlighted that while secondary or incidental findings should be included within analyses, they were often associated with high levels of uncertainty and excluded from the scope of analyses. Whilst the literature [9,18,22] highlights the importance of comparator choice, there is little structured discussion about the broader aspects of the scope. None of the literature framed the discussion from a complex intervention paradigm, and specifically whether such a position would assist in meeting the scoping challenge raised by the above interactions.

#### 3.2.2. Defining the Model Structure

**Figure 3:**
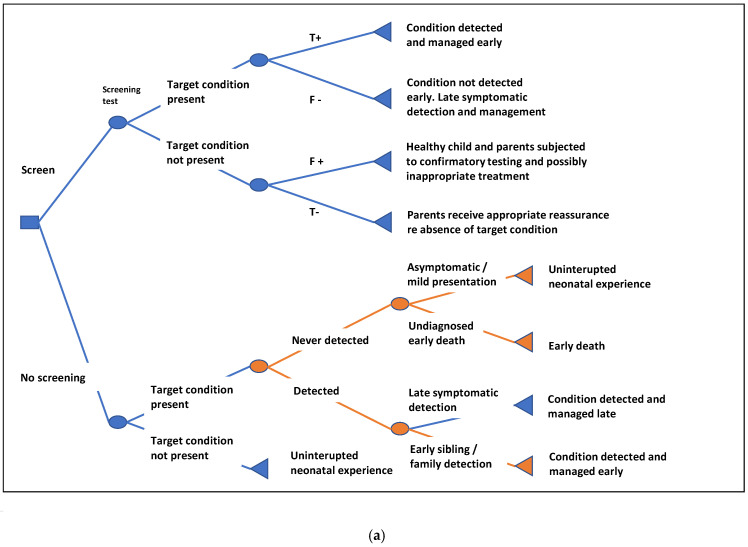

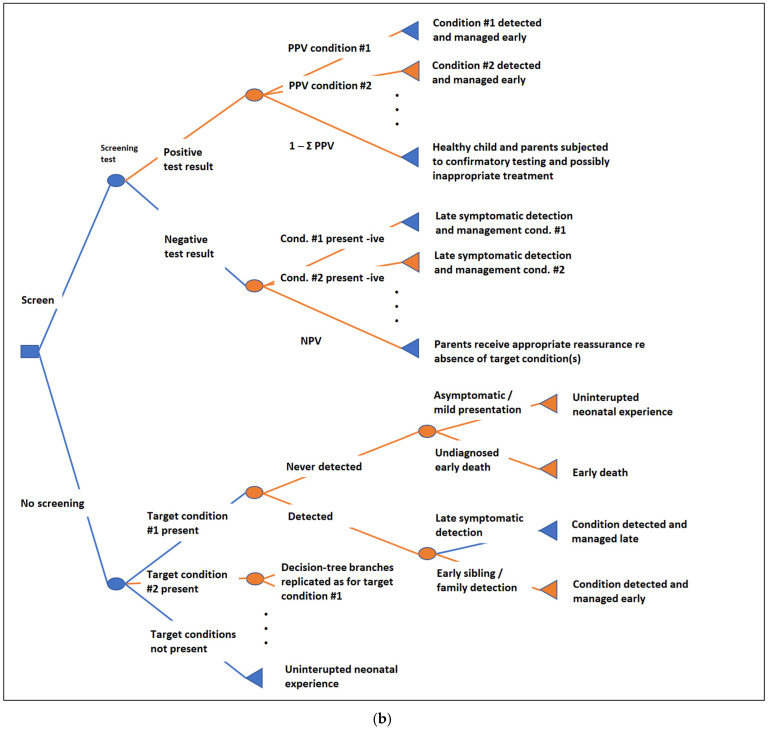
Optional structures for the NBS decision problem (shown in orange): (**a**) Expanding the no screening arm; (**b**) Incorporating multiple target conditions.

The simple NBS model structure described in Figure 1 represents an a priori framework for data extraction and discussion in this study, not a recommended NBS model structure. Only two studies explicitly discussed the definition of model structure [22,23]. Analysts within the group suggested that alternative structures may be appropriate, for instance, extensions to account for issues such as family history detection; biases in symptomatic incidence evidence from either under-ascertainment or asymptomatic or mild presentations (Figure 3a); or the case where multiple conditions are detected (Figure 3b).

#### 3.2.3. Selecting the Modelling Method

The most common modelling method implemented for NBS is the decision trees that present decisions and chance events in the order that they occur, with Markov models being used to estimate long-term outcomes in a subset and with patient level models only used occasionally [5,8,9]. In their review of the evidence, Karnon identified no comparative methodological studies [5]; further review by Cacciatore suggested the choice of modelling approach was often not substantiated [9], highlighting that there may be scope to clarify the options available and the pros, cons, and circumstances that underpin preferred choices.

### 3.3. The Target Condition

This aspect of the decision problem concerns estimating the incidence of target conditions. In addition to issues associated with rarity, biases in observational data, such as ascertainment, referral, selection, and condition-spectrum biases, can present challenges [10,16,17,19]. For example, observational data may not include cases where there was a death prior to diagnosis or milder cases that may be identified by screening. The use of well-structured cross-national registries and, where feasible, retrospective testing of dried bloodspots (DBS) may offer approaches to mitigating these biases [18,20].

The importance of having an a priori case definition for a target condition is well recognised [30,31]. Notwithstanding this, experience from screening evaluations, for example, in screening for severe combined immunodeficiency (SCID), suggests that case definitions are subject to evolution. This can arise where screening test findings in practice are wider than the reported evidence, meaning that clinicians must respond to account for these other conditions or variants. This aspect of complexity impacts on the ability to estimate both incidence and outcomes.

Workshop discussions highlighted that it is also necessary to understand the timing of disease presentation, as this could determine the cases identified by screening and actionability. Furthermore, as understanding of the genetic underpinnings of a target condition increases, it may be necessary to identify genetic subgroup incidence, especially where this impacts on management and outcomes.

### 3.4. The Screening Test/Protocol

This section focuses on challenges arising in modelling screening test characteristics and diagnostic protocol impacts. Firstly, there is no consistent terminology for referring to conditions that might be identified by screening beyond the primary target. Many terms have been used, including ‘secondary target’, ‘incidental/unintended findings’, ‘ambiguous results’, and ‘overdiagnosis’ [32]. Furthermore, the range of screening impacts on parents and carers has been associated with the term ‘spillover effects’. However, the workshop discussion indicated that, notwithstanding an emerging consensus within the health economics community [33], the scope of impacts implied by this term was not commonly understood. Therefore, there remains a challenge in clarifying terminology in the NBS domain, which hampers evidence synthesis and appropriate clinical and user involvement.

Early evidence on screening test characteristics, for example, sensitivity and specificity, is commonly based on case–control or two-gate designs, with the cases and controls drawn from separate populations that are subject to methodological bias [34]. Furthermore, where screening has been introduced, test accuracy data are often not published or determined through systematic follow-up.

Historically, policy making has focused on decision making at the target condition level, with technologies being introduced following evaluation with a limited number of conditions. For example, in the UK and in Washington (US), tandem mass spectrometry was introduced with the addition of screening for medium chain acyl-CoA dehydrogenase deficiency [17,35]. Subsequent expansion of screening for inborn errors of the metabolism [36] benefitted from a low marginal cost of testing. Workshop discussion highlighted that emerging genomic screening technologies may challenge this single condition approach to decision making and economic evaluation. Png, Wright, and Cacciatore [8,9,19] found that few studies have considered the cost or health impacts of screening tests on parents/carers, including the impact of information provision and genetic testing. Castilla-Rodríguez et al. highlighted the potential impacts of parental reproductive choice on the evolution of disorder incidence and that incorporating such multi-cohort effects would be a significant methodological challenge [20]. Whilst quality-of-life instruments are inadequate for capturing many parental impacts [21], assessments using time trade-off or willingness-to-pay methods have been used [37,38]. Ulph et al. further demonstrated that the mode and quality of information provision can moderate the potential negative effects of false positive results emphasising a further aspect of the complexity of the newborn screening intervention [24].

Both the literature and workshop highlighted the potential for screening to simplify the path to a definitive diagnosis, the ‘diagnostic odyssey’. However, where these effects are incorporated in economic evaluations, analyses often rely upon observational evidence that can be biased- [16]. Furthermore, few economic evaluations address impacts for those with false positive or non-target findings [8].

### 3.5. Outcome Nodes

Challenges were identified in the literature and workshop discussions concerning the selection, measurement and valuation of the cost, resource, and health effects relevant to the outcome nodes in the NBS model. Png [8] identified that there was no consistency in the selection of these outcomes in economic evaluations and, particularly, that few studies included outcomes from overdiagnosis and spillover effects. Furthermore, outcomes associated with the diagnosis of ambiguous or later-onset cases are often excluded [16].

In the absence of randomised controlled comparisons, there is a heavy reliance on historical or contemporary observational evidence to estimate screening effects; however, such data are subject to many biases [11,18,20,22]. Furthermore, data on long-term outcomes, quality of life, and cost impacts of a condition are often incomplete or missing, even for conventional management [18]. The workshop discussion highlighted that these issues are compounded when screening changes the disease classification system, for example, from a phenotype-based system (based on age and severity of symptoms) to a genetic-based system (based upon absence or presence of genes, many of which can be of uncertain clinical significance).

Challenges in the measurement and valuation of quality of life in children are well documented [5,15,39,40,41]. Both the literature and workshops highlighted that the young age of children and the rarity of conditions made this a particular issue in NBS. In some economic studies, this has led to a cost-per—life-year approach being used, which results in the exclusion of morbidity benefits or harms from screening. It was suggested in the workshop that measuring and valuing the effects of early intervention over the life course, for instance, “health in a neonate, in a teenager, to health in a young adult, to adult health through to the end of life”, throws up a fundamental challenge, over and above the problems at any individual life stage.

### 3.6. Other Considerations

The literature [18,22] and workshop discussion consistently identify the challenge that high levels of uncertainty throughout the NBS problem pose for decision making. A key challenge for analysts and policy makers is to develop methods and processes for characterising uncertainty across the range of outcomes identified as necessary to support NBS decision making.

Workshop discussions also highlighted that the systemic nature of NBS offers an opportunity for taking a structured approach to evidence generation. For instance, in the UK, the existence of a nationally organised screening service feeding into the universal NHS services for management and treatment provides significant opportunities for an iterative approach to evidence generation, economic modelling, and evidence-based policy and practice [18,22].

### 3.7. Recommendations Regarding Decision Processes and Methods Research

#### 3.7.1. Supporting NBS Decision Making

Box 1 presents the recommendations for supporting NBS decision making. The review and workshop discussions identified a need for guidance on the range of health economic, health, resource, and cost outcomes that are required to adequately support NBS decision making. It is proposed that the production of a defined outcome set may enable different perspectives to be addressed, enable short- and long-term health, resource, and cost outcomes to be assessed, and enable the impact of differential uncertainties to be examined. Since NBS interventions impact the provision of downstream healthcare services, this outcome set should support coherent decision making along the healthcare pathway.

Box 1 **Recommendations for supporting NBS decision making.**

Process: Health economics support to NBS decision makers should entail the production of a set of outcomes that includes both summative measures of economic performance, such as the incremental cost-effectiveness ratio, and disaggregated measures of costs, health, and resource consequences.

* *

Research: Health economists and NBS decision makers should collaborate in research to define and test a set of cost and consequence outcomes that would support NBS policy making.

* *

#### 3.7.2. NBS Model Structure and Methods

The recommendations regarding model structure and methods are presented in Box 2. The workshops highlighted a lack of a consistently understood terminology that hinders NBS decision modelling. Development is required to establish NBS terminology, addressing concepts, including target condition, secondary target, incidental finding, false positive, and spillover effect.

The development of modelling guidance that clarifies the design questions to be addressed in specifying an NBS decision model would benefit the NBS decision support community. Good practice in model development requires an iterative and parsimonious process, which stops when a proper validity-complexity trade-off is achieved. Well-designed guidance may contribute to shortening iterations and enhance the validity, comparability, and credibility of models. NBS models include at least two well-differentiated submodels: the disease treatment submodel, subject to the same guidance as any other disease, rare or not, and the screening process/clinical detection submodel. NBS model development would benefit from a series of structured questions addressing both domains, such as those in Table 2.

The workshop discussion demonstrated a broad agreement that NBS is a complex intervention. There is an opportunity to examine the relevance and implications of current developments in complex intervention evaluation for NBS decision making processes [2,42] and the implications for health economic methods. For instance, to what extent can NBS decision models provide the programme theory required to underpin iterative evaluation and implementation and to what extent are complex system modelling methods required to adequately capture the economic impact of screening [43]? With regard to iterative evaluation, what are the implications for evidence infrastructure, and can post-implementation surveillance mechanisms be used to determine therapy effectiveness, for instance by bringing a ‘managed access’ approach to screening programmes?

Box 2 **Recommendations for NBS model structure and methods.**

Research: Development of an NBS terminology and guidance on NBS model design and the design questions arising. Modelling case studies to test terminology and modelling guidance in implementation.

* *

Research: Critically examine the relevance and implications of current guidance on complex intervention evaluation for NBS decision-making processes and the implications for health economic decision support needs and methods. Note this may also have implications for decision processes and evidence infrastructure.

* *

#### 3.7.3. Data and Estimation of Model Parameters

Weaknesses in the evidence base, including the lack of good quality and unbiased data on essentially all aspects of the NBS decision problem, are a major theme in the methodological literature and workshop discussions. Given the broad scope of these challenges, this discussion considers that a structured approach is required to better define evidence needs, assess data collection feasibility, and identify and assess current evidence sources for data availability, quality, and bias. The recommendations for data and estimation of model parameters are included in Box 3.

Research into a common description of parameters required in the development of an NBS decision model and guidance on model design would improve consistency and credibility in NBS economic evaluations. This research could build upon the ideas developed by Prieto-Gonzalez et al. [23] and may examine the feasibility of extending this parameter set to define a formal NBS decision modelling ontology to help bridge the gap between data sources and policy information requirements. Such a framework could also benefit evidence maps [44] and reviews, improving methods and consistency. Where data collection is not feasible, researchers could propose proxy evidence or alternative model structures. Where data collection is constrained by current methods, this may inform NBS methodological research priorities, for instance in measuring and valuing parental/carer and child outcomes.

A systematic examination of NBS relevant data availability and quality would be beneficial. In the UK, routinely collected data on healthcare resource usage for rare disease management is potentially available through sources such as Hospital Episode Statistics in England and the Congenital Anomaly Register and Information Service in Wales. These data are currently underutilised in economic evaluations of NBS; initiatives to improve access, linkage, and usability of these resources should be encouraged. Similarly, a systematic approach to examining the data availability and quality of condition-specific registries and other observational evidence sources to support NBS decision modelling would be beneficial. As an initial step, research could focus on a small number of health conditions to examine sources and make recommendations on scope and quality of data collection, both within the particular registries concerned and more generically. Policy makers should seek ways to support the development of evidence infrastructure, for instance, disease registries, systematic approaches to DBS storage and usage, and international collaboration, with a special focus on ensuring that evidence is sufficient to support economic evaluation.

Much of the evidence available to support decision making is subject to bias, especially observational evidence. There are two broad analytical approaches available, firstly the use of methods to account and adjust for bias and secondly sensitivity analysis to estimate the potential importance of bias for decision uncertainty. Research is required to explore the feasibility of analytical approaches to mitigate bias in estimates of key NBS model parameters such as incidence, disease progression, and outcomes. Examples of approaches may include the use of subjective judgement within meta-analysis [20], causal inference methods, or Bayesian model calibration approaches [5].

Box 3 **Recommendations for data and estimation of model parameters.**

Research: Define a description of parameters required in the building of an NBS decision model. Assess the feasibility of data collection, devise alternative proxy evidence and model structures, and identify methods requirements.

* *

Process: Improve access and usability of routine data sources for providing evidence for NBS economic assessment.

* *

Research: Collaborative case study research to review the scope and quality of data available within a small number of condition-specific data sources to support NBS decision modelling. It is suggested that health conditions examined should display a range of rarities and include novel and existing screening interventions.

* *

Research: Explore analytical approaches, including, for instance, causal inference or model calibration methods, to deliver unbiased estimates of key NBS model parameters.

* *

#### 3.7.4. Overarching Recommendations

Box 4 presents the overarching recommendations. Workshop discussion identified uncertainty as a key challenge for NBS decision modelling, affecting both the structure and parameterisation of a model and its credibility for decision support. The above research on model design seeks to minimise structural uncertainty and provide the basis for defining alternative model structures that can be explored in scenario analyses. Guidelines on parametric uncertainty analysis in health economic models focus on describing uncertainty in cost-effectiveness [45]. Research is required in exploring methods of presenting uncertainty in cost-consequence outcome sets in a form helpful to NBS decision makers.

A health economics reference case is a set of recommended methodological practices that seeks to improve the quality and comparability of analyses available to support decision making [46,47,48]. The scope of a reference case includes definition of decision variables, for example, perspective and discounting, outcomes of interest, modelling methods, and methods for outcome measurement and valuation. Many of the recommendations presented above would be relevant to the development of an economic reference case and could provide the basis for policy makers to consider an NBS reference case for their jurisdiction.

NBS is a complex intervention bringing together stakeholders from the diagnostics, therapy development, and behavioural interventions communities. This study has focused on the challenges arising for NBS policy making at the end of the intervention development pipeline. Meeting these evidential challenges may, however, require collaboration between the aforementioned stakeholders drawn from the academic, commercial, and patient voice sectors. Examples of good collaborative practice in the rare disease setting are provided by recent work in Duchenne muscular dystrophy [49,50,51]. Initiatives to support such international collaboration in the NBS domain are recommended, for instance, in developing early-stage natural history models, development of outcomes measures and instruments focused on patient spillover effects, and research on the performance of tests in the asymptomatic population.

Box 4 **Overarching Recommendations.**

Research: Explore different methods of presenting uncertainty in cost-consequence analyses in a form helpful for NBS decision makers.

* *

Process: NBS policy makers should consider the benefits and drawbacks of making their decision support needs explicit through the development of a health economics NBS reference case for their jurisdiction.

* *

Process: Initiatives to support international collaboration between academic, commercial, and parent/patient support groups in the NBS domain that include the development of economic evidence infrastructure, for instance, early-stage natural history models, outcomes measures and instruments, including patient spillover effects, and research on test performance in asymptomatic populations.

* *

## 4. Discussion and Conclusions

Guidance on methods reviews is underdeveloped [12]. This study used an iterative pearl growing approach to study identification, with initial grit studies based on the BSTG’s project specification. Other starting points may have resulted in a different set of literature. In mitigation, the protocol specified saturation as the stopping criteria for searching, and this was achieved with only one further study being suggested by experts at the workshop stage.

The inclusion/exclusion criteria specified papers addressing health economics of NBS; this meant that relevant discussions may have been excluded. For example, whilst studies exist concerning challenges in economic evaluation of genetic testing [52,53], there are few that explicitly address NBS. Ongoing research in genetic NBS [54,55] means that considering these challenges is of high priority.

Regarding data extraction, the narrative nature of the modelling challenge descriptions gave rise to some differences in text extraction between the two reviewers. There was, however, a high level of agreement in issues identified, and topic coding was generally in agreement. Feedback from workshops concerning the topic framework was also positive.

The original protocol had specified a systematic review of NBS case studies to accompany the methods review. The recent systematic reviews of NBS models identified in the methods review meant that this was considered superfluous; the protocol was, therefore, modified to allow case studies to emerge directly from the methodological discussion.

The project specification was about identifying solutions. The nature of the challenges identified, however, means that many of the recommendations are about finding a pragmatic way forward, involving collaborative research to further define and develop solutions. For instance, the need for international collaboration in developing rare disease evidence is well recognised. However, in reviewing European rare disease data, Prieto-Gonzalez et al. [23] demonstrated that opportunities to collect economically relevant data are being missed. Solving this issue will require collaborative initiatives between health economists and the rare disease community.

The theory and practice of health economics are in continual development, with debate on the welfarist, extra-welfarist, or non-welfarist bases; the role of maximisation as a policy aim; and methods for the measurement and valuation of health and non-health impacts, for instance through capabilities or extensions of the QALY [56,57]. The recommendation for the production of a set of cost and consequence outcomes to enable NBS policy making that reflects current decision-making practice is a pragmatic approach to decision support that has the potential to evolve in line with developments in theory and methods.

## Figures and Tables

**Table 1:** Critical appraisal and data extraction summary.

Study	SANRA Score	Decision Criteria	Decision Variables	Decision Problem Scope	Defining Model Structure	Selecting Modelling Method	The Target Condition	The Screening Test/Protocol	Outcome Nodes	Other
Cacciatore et al., 2020 [9]	12		√	√		√		√	√	√
Castilla Rodriguez et al., 2017 [20]	8					√	√	√	√	√
Grosse et al., 2007 [14]	7			√					√	√
Grosse et al., 2009 [21]	8	√	√							
Grosse et al., 2010 [15]	3								√	
Grosse et al., 2015 [16]	9							√	√	
Grosse et al., 2016 [17]	10						√		√	
Grosse et al., 2020 [18]	9		√	√			√		√	√
Ho et al., 2023 [11]	5	√					√		√	
Karnon et al., 2007 [5]	11					√		√	√	√
Langer et al., 2012 [6]	12	√							√	√
Png, 2022 [8]	12	√	√					√	√	
Prieto-González et al., 2019 [23]	12				√					
Prosser et al., 2012 [22]	10	√	√	√	√				√	√
Ulph et al., 2017 [24]	12							√		√
Wright et al., 2015 [19]	10							√		

**Table 2:** Examples of design questions for model developers.

Question	Decision Affected
Does the heterogeneity of patients’ attributes affect the outcomes in a non-linear fashion?	Modelling approach
Does the screening/diagnosis protocol include complex strategies that depend on individual patient characteristics?	Modelling approach
Does a positive test result involve conditions other than the target?	Model structure
Is the screening strategy a complex intervention, which comprises a series of tests, recalls, etc., that have a remarkable impact on outcomes?	Model structure
Is there evidence that ascertainment bias plays a critical role in the affected population?	Model structure

## Data Availability

The systematic review component of this research relies on publicly available data. The workshop data is available on request from the corresponding author.

## Supplementary Materials

The following supporting information can be downloaded at: https://www.mdpi.com/article/10.3390/ijns11040096/s1, Supplementary Material: Review Methods; Glossary of NBS terms for decision modelling.

## Abbreviations

The following abbreviations are used in this manuscript:

BSTG	Blood Spot Task Group of the UKNSC
DBS	Dried Bloodspot
DHSC	Department of Health and Social Care
HIQA	Health Information and Quality Authority
NBS	Newborn Bloodspot Screening
OHID	Office of Health Improvement and Disparities
SANRA	Scale for the Assessment of Narrative Review Articles
SCHARR	Sheffield Centre for Health and Related Research
SCID	Severe Combined Immunodeficiency
UKNSC	UK National Screening Committee
