# Supplementary material for: Methodological and Procedural Considerations for Developing Decision Analytic Models to Assess the Health Economic Impacts of Newborn Bloodspot Screening: A Systematic Methodological Review
Source: Int J Neonatal Screen. 2025 Oct 17;11(4):96. doi: 10.3390/ijns11040096 (PMC12550984; doi:10.3390/ijns11040096)
Supplement: Supplementary file 1 [file IJNS-11-00096-s001.zip › IJNS-3727004-supplementary.docx]

**Title: Methodological and procedural considerations for developing decision analytic models to assess the health economic impacts of newborn bloodspot screening: A systematic methodological review**

**SUPPLEMENTARY MATERIAL**

**CONTENTS**

SUPPLEMENTARY MATERIAL 1 – REVIEW METHODS 2

SUPPLEMENTARY MATERIAL 2 – GLOSSARY OF NBS TERMS FOR DECISION MODELLING 11

**SUPPLEMENTARY MATERIAL 1 – REVIEW METHODS**

**Search Methods**

Searches were conducted in July-August 2023. A pearl-growing^1^ iterative approach was utilised due to the known challenges of searching for methodological reviews^2^. A core set of “pearls” (five studies^3-7^) was identified and agreed with the NSC evidence team. The indexing and metadata of these pearls was examined and used to construct a search strategy for Ovid MEDLINE. This search strategy was run for iteration 1 and retrieved 213 references. The titles and abstracts of these references were screened to inform iteration 2 of the search.

**Iteration 2**

The 30 included studies from Iteration 1 were examined to identify the indexing and metadata that had retrieved them. MeSH headings and search terms that did not uniquely identify any included studies were removed from the search strategy for Iteration 2. In addition, some terms were added or edited to broaden their retrieval based on the included studies. For example “cost-effectiveness analyses” was added, methodological was changed to methodolog* and the proximity operator changed to adj5. Limitations was added to the terms proceeding methodolog*. This adapted search strategy was then re-run in Ovid MEDLINE, and combined with Iteration 1 using the operator NOT, to identify 4 additional unique references. The iteration 2 search strategy was then translated and conducted on EMBASE via Ovid and EconLit via Ovid. TUFTS CEA registry and MATHSCINET were searched using the keyword “newborn screening” due to the lack of functionality to create systematic search strategies.

**Iteration 3**

For iteration 3 (October 2023), two new pearls (included studies) were identified^8-9^. A further two MeSH headings were identified as relevant, and five EMTREE headings. Focused searches were run on MEDLINE and Embase to incorporate these further terms. No new included studies were identified from iteration 3, so the searching ceased at this point as it implied that retrieval saturation had been reached.

**Inclusion & exclusion criteria**

***Inclusion criteria***

Methodological papers dealing with processes and methods for undertaking economic decision models of newborn bloodspot screening interventions.

***Exclusion criteria***

Health economic model reports evaluating screening interventions in a single application (Exclude - Case study).

Reviews of economic models of a single screening intervention (Exclude – Case study).

Methods papers not concerning health economics (Exclude – Not HE).

Methods papers not concerning newborn screening (Exclude – Not NS).

Note health economics methods papers concerning diagnostic test evaluation identified in the search were included at the title and abstract stage, but excluded at full text stage if the text did not refer to the newborn screening context.

**Data extraction and coding**

Data extraction and coding is available from the authors on request.

**Review methods references**

1. Schlosser, R. W., Wendt, O., Bhavnani, S., & Nail‐Chiwetalu, B. (2006). Use of information‐seeking strategies for developing systematic reviews and engaging in evidence‐based practice: the application of traditional and comprehensive Pearl Growing. A review. *International Journal of Language & Communication Disorders*, *41*(5), 567-582. DOI: <https://doi.org/10.1080/13682820600742190>
2. Lilford, R. J., Richardson, A., Stevens, A., Fitzpatrick, R., Edwards, S., Rock, F., & Hutton, J. L. (2001). Issues in methodological research: perspectives from researchers and commissioners. *Health Technology Assessment (Winchester, England)*, *5*(8), 1-57. DOI: [10.3310/hta5080](https://doi.org/10.3310/hta5080)
3. Png, M.E., Yang, M., Taylor-Phillips, S., Ratushnyak, S., Roberts, N., White, A., Hinton, L., Boardman, F., McNiven, A., Fisher, J. and Thilaganathan, B., 2022. Benefits and harms adopted by health economic assessments evaluating antenatal and newborn screening programmes in OECD countries: A systematic review of 336 articles and reports. *Social Science & Medicine*, p.115428. DOI: <https://doi.org/10.1016/j.socscimed.2022.115428>
4. Karnon, J., Goyder, E., Tappenden, P., McPhie, S., Towers, I., Brazier, J., & Madan, J. (2007). A review and critique of modelling in prioritising and designing screening programmes. *HEALTH TECHNOLOGY ASSESSMENT-SOUTHAMPTON-*, *11*(52). DOI: <https://doi.org/10.3310/hta11520>
5. Cacciatore, P., Visser, L. A., Buyukkaramikli, N., van der Ploeg, C. P., & van den Akker-van Marle, M. E. (2020). The methodological quality and challenges in conducting economic evaluations of newborn screening: a scoping review. *International Journal of Neonatal Screening*, *6*(4), 94. DOI: <https://doi.org/10.3390/ijns6040094>
6. Langer, A., Holle, R., & John, J. (2012). Specific guidelines for assessing and improving the methodological quality of economic evaluations of newborn screening. *BMC health services research*, *12*(1), 1-12. DOI: <https://doi.org/10.1186/1472-6963-12-300>
7. Phillips, K. A., Deverka, P. A., Marshall, D. A., Wordsworth, S., Regier, D. A., Christensen, K. D., & Buchanan, J. (2018). Methodological issues in assessing the economic value of next-generation sequencing tests: many challenges and not enough solutions. *Value in Health*, *21*(9), 1033-1042. DOI: <https://doi.org/10.1016/j.jval.2018.06.017>
8. Castilla-Rodríguez, I., Vallejo-Torres, L., Couce, M. L., Valcárcel-Nazco, C., Mar, J., & Serrano-Aguilar, P. (2017). Cost-effectiveness methods and newborn screening assessment. *Rare Diseases Epidemiology: Update and Overview*, 267-281. DOI: <https://doi.org/10.1007/978-3-319-67144-4_16>
9. Van Ho, H., Giguère, Y., & Reinharz, D. (2023). Expected Benefits and Challenges of Using Economic Evaluations to Make Decisions About the Content of Newborn Screening Programs in Vietnam: A Scoping Review of the Literature. *Journal of Inborn Errors of Metabolism and Screening*, *11*, e20220011. DOI: <https://doi.org/10.1590/2326-4594-JIEMS-2022-0011>

**Iteration 1 Search Strategy (conducted on MEDLINE only 31 July 2023)**

Ovid MEDLINE(R) and Epub Ahead of Print, In-Process, In-Data-Review & Other Non-Indexed Citations and Daily <1946 to July 30, 2023>

1 *Neonatal Screening/ 7419

2 newborn screening.ti,ab. 7254

3 *Genetic Diseases, Inborn/di [Diagnosis] 1910

4 *Genetic Testing/ec [Economics] 552

5 Genetic Testing/mt [Methods] 11422

6 High-Throughput Nucleotide Sequencing/ 46013

7 or/1-6 70120

8 Mass Screening/ec [Economics] 6530

9 *Mass Screening/og [Organization & Administration] 2464

10 8 or 9 8852

11 Child/ 1916856

12 Child Health Services/og [Organization & Administration] 5524

13 Infant, Newborn/ 670192

14 11 or 12 or 13 2411391

15 10 and 14 944

16 7 or 15 71016

17 Cost-Benefit Analysis/ 92810

18 Decision Support Techniques/ 22487

19 "Costs and Cost Analysis"/ 51444

20 health economic assessment*.ti,ab. 169

21 screening model*.ti,ab. 2207

22 (modelling adj (methods or techniques or approach*)).ti,ab. 8160

23 (cost-effectiveness analysis or CEA).ti,ab. 38401

24 (cost-utility analyses or CUA).ti,ab. 2226

25 economic evaluation*.ti,ab. 15083

26 Technology Assessment, Biomedical/ 11038

27 or/17-26 215218

28 16 and 27 1692

29 (review or critique or critical appraisal).ti,ab. 2164837

30 (methodological adj (study or studies or quality or challenges or issues)).ti,ab. 36195

31 (assess* adj3 quality).ti,ab. 101841

32 or/29-31 2236670

33 28 and 32 213

**Iteration 2 Search Strategy**

**MEDLINE 2 August 2023**

Ovid MEDLINE(R) and Epub Ahead of Print, In-Process, In-Data-Review & Other Non-Indexed Citations and Daily <1946 to August 01, 2023>

1 *Neonatal Screening/ 7419

2 newborn screening.ti,ab. 7254

3 *Genetic Diseases, Inborn/di [Diagnosis] 1910

4 *Genetic Testing/ec [Economics] 552

5 Genetic Testing/mt [Methods] 11422

6 High-Throughput Nucleotide Sequencing/ 46013

7 or/1-6 70120

8 Mass Screening/ec [Economics] 6530

9 Child/ 1916856

10 Infant, Newborn/ 670192

11 9 or 10 2410856

12 8 and 11 656

13 7 or 12 70743

14 Cost-Benefit Analysis/ 92810

15 "Costs and Cost Analysis"/ 51444

16 cost-effectiveness analysis.ti,ab. 12307

17 cost-effectiveness analyses.ti,ab. 3127

18 economic evaluation*.ti,ab. 15083

19 Technology Assessment, Biomedical/ 11038

20 or/14-19 159899

21 (review or critique).ti,ab. 2160174

22 (methodolog* adj5 (quality or challenges or limitations)).ti,ab. 42989

23 21 or 22 2174868

24 13 and 20 and 23 190

**EMBASE via Ovid 3 August 2023**

Embase <1974 to 2023 Week 30>

1 *newborn screening/ 9905

2 newborn screening.ti,ab. 11875

3 *genetic disorder/di [Diagnosis] 4752

4 *genetic screening/ 21593

5 ec.fs. 6484972

6 4 and 5 5817

7 genetic screening/ 115424

8 methods.kw. 4745

9 7 and 8 7

10 high throughput sequencing/ 91718

11 1 or 2 or 3 or 6 or 9 or 10 117589

12 mass screening/ 61484

13 5 and 12 4392

14 child/ 2086076

15 newborn/ 600422

16 14 or 15 2570655

17 13 and 16 502

18 11 or 17 118033

19 "cost benefit analysis"/ 94401

20 "cost"/ 63021

21 cost-effectiveness analysis.ti,ab. 18918

22 cost-effectiveness analyses.ti,ab. 4299

23 economic evaluation*.ti,ab. 21263

24 biomedical technology assessment/ 17077

25 19 or 20 or 21 or 22 or 23 or 24 203749

26 (review or critique).ti,ab. 2640433

27 (methodolog* adj5 (quality or challenges or limitations)).ti,ab. 51764

28 26 or 27 2659323

29 18 and 25 and 28 138

**EconLit via Ovid 3 August 2023**

Econlit <1886 to July 27, 2023>

1 Neonatal Screening.mp. [mp=heading words, abstract, title, country as subject] 1

2 newborn screening.ti,ab. 7

3 genetic diseases.mp. [mp=heading words, abstract, title, country as subject] 5

4 genetic testing.mp. [mp=heading words, abstract, title, country as subject] 101

5 High-Throughput Nucleotide Sequencing.mp. [mp=heading words, abstract, title, country as subject] 0

6 1 or 2 or 3 or 4 or 5 113

7 Mass Screening.mp. [mp=heading words, abstract, title, country as subject] 8

8 Child*.mp. [mp=heading words, abstract, title, country as subject] 46057

9 (newborn* or infant*).mp. [mp=heading words, abstract, title, country as subject] 3365

10 8 or 9 47403

11 7 and 10 0

12 6 or 11 113

13 Cost-Benefit Analysis.mp. [mp=heading words, abstract, title, country as subject] 8713

14 "Costs and Cost Analysis".mp. [mp=heading words, abstract, title, country as subject] 2

15 cost-effectiveness analysis.mp. [mp=heading words, abstract, title, country as subject] 650

16 cost-effectiveness analyses.mp. [mp=heading words, abstract, title, country as subject] 137

17 economic evaluation*.mp. [mp=heading words, abstract, title, country as subject] 1633

18 technology assessment.mp. [mp=heading words, abstract, title, country as subject] 320

19 13 or 14 or 15 or 16 or 17 or 18 10725

20 12 and 19 14

21 (review or critique).mp. [mp=heading words, abstract, title, country as subject] 54603

22 (methodolog* adj5 (quality or challenges or limitations)).mp. [mp=heading words, abstract, title, country as subject] 1038

23 21 or 22 55479

24 20 and 23 4

**TUFTS CEA Registry 4 August 2023**


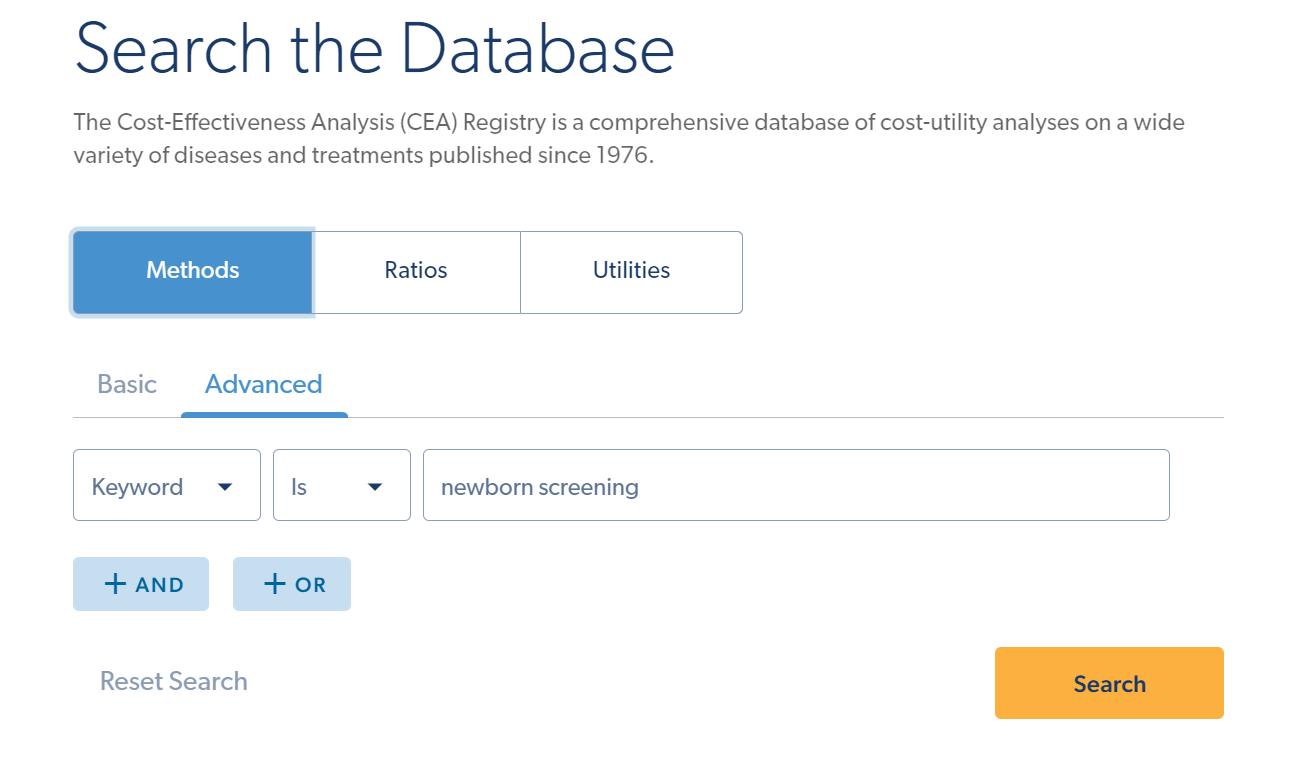


**MATHSCINET 7 August 2023**


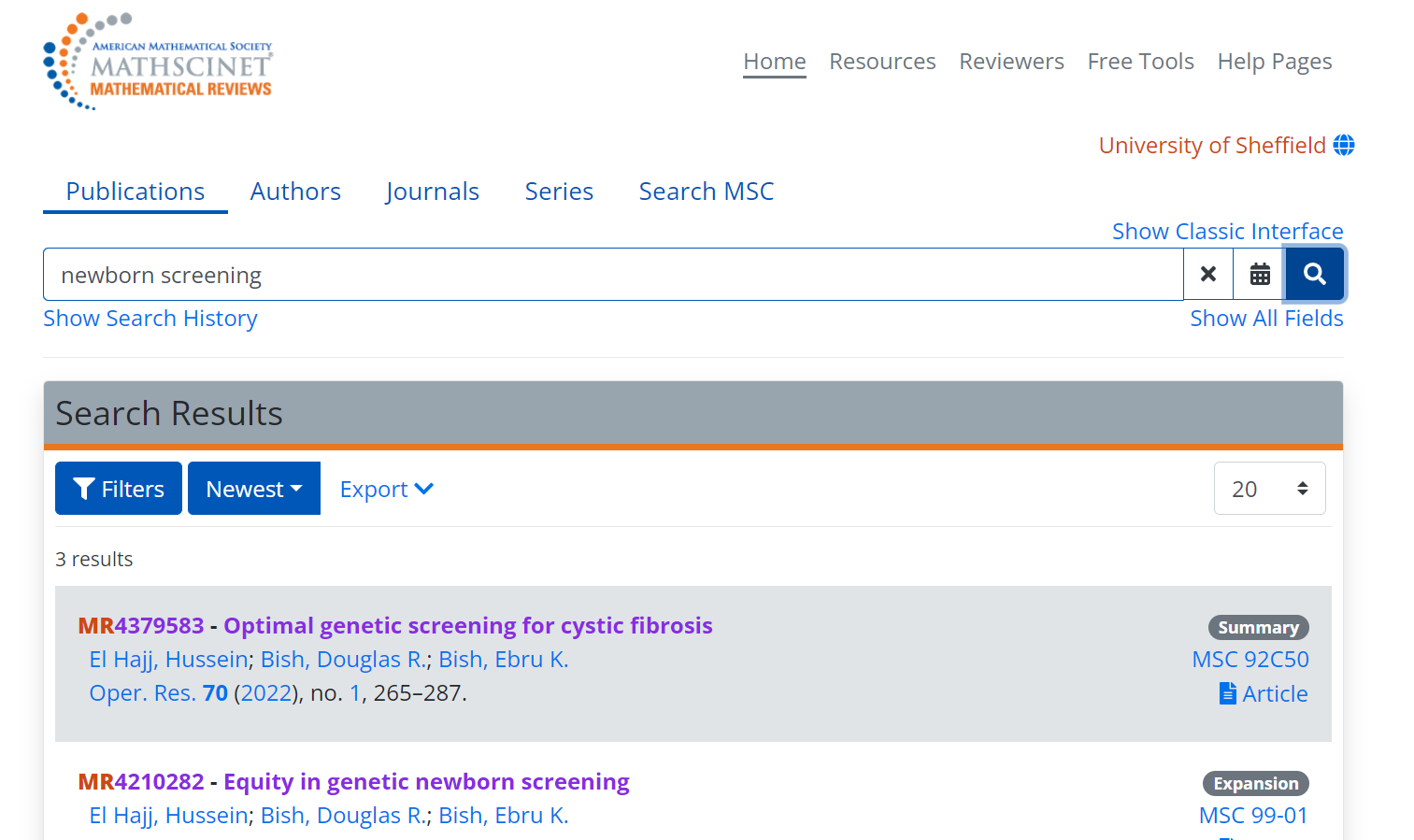


**Iteration 3 Search Strategy**

**MEDLINE 27 October 2023**

Ovid MEDLINE(R) and Epub Ahead of Print, In-Process, In-Data-Review & Other Non-Indexed Citations and Daily <1946 to October 26, 2023>

1 *Neonatal Screening/ 7432

2 newborn screening.ti,ab. 7403

3 *Genetic Diseases, Inborn/di [Diagnosis] 1910

4 *Genetic Testing/ec [Economics] 552

5 Genetic Testing/mt [Methods] 11453

6 High-Throughput Nucleotide Sequencing/ 46399

7 or/1-6 70681

8 *Health Care Costs/ 20625

9 Models, Economic/ 11088

10 Quality-Adjusted Life Years/ 15870

11 8 or 9 or 10 43762

12 7 and 11 219

13 (review or critique).ti,ab. 2199449

14 (methodolog* adj5 (quality or challenges or limitations)).ti,ab. 43890

15 13 or 14 2214382

16 12 and 15 26

**EMBASE via Ovid 27 October 2023**

Embase <1974 to 2023 Week 42>

1 *newborn screening/ 10051

2 newborn screening.ti. 5239

3 (review or critique).ti,ab. 2688307

4 (methodolog* adj5 (quality or challenges or limitations)).ti,ab. 52725

5 3 or 4 2707499

6 *decision making/ 70377

7 *economic aspect/ 16502

8 health economics/ 35975

9 outcome assessment/ 872906

10 "quality of life"/ 629742

11 economic model/ 3289

12 "health care cost"/ 228200

13 or/6-12 1741486

14 1 or 2 10644

15 5 and 13 and 14 128

**SUPPLEMENTARY MATERIAL 2 – GLOSSARY OF NBS TERMS FOR DECISION MODELLING**

The following glossary of terms is provided as an exemplar. The scope of the glossary is not intended to be complete, and the definitions provided are draft. This is provided as a starting point for further research on NBS screening modelling terminology.

**Table S1 Glossary of NBS terms for decision modelling**

| **Term** | **Definition** |
| --- | --- |
| Primary target | Prospectively defined actionable target health condition identified by the screening protocol. |
| Secondary target | Prospectively defined actionable health condition that may also be identified by the screening protocol. |
| Incidental finding | A health condition identified by screening which is not a prospectively defined target in the screening protocol. |
| Actionable | There is an evidence-based screening pathway that directly improves the health outcomes of the screened newborn. |
| Screening pathway | Systematic end-to-end quality-assured process from inviting individuals for screening to intervention. |
| Screening protocol | Screening test definitions, confirmatory tests, diagnostic protocols and guidance for management and treatment of target conditions. |
| Screening test | Evidence-based initial test with the goal of identifying individuals at risk for a target health condition. |
| Confirmatory test | Diagnostic test triggered by a screen positive result. |
| Diagnostic protocol | Guidance and protocols to ensure effective clinical diagnosis. |
| Ascertainment bias | Bias affecting estimates of incidence that may also influence outcome assessment. |
| Spillover effects | Unintended consequences of the screening pathway that can impact stakeholders, both positively and negatively. |
